# Nursing Education in Catalonia: Novice Professionals’ Appraisal of Its Quality and Usefulness. Does Mobility Play a Role?

**DOI:** 10.3390/ijerph17197145

**Published:** 2020-09-29

**Authors:** Teresa Peiró, Beatriz Sora, Aida Soriano, Jesús Yeves

**Affiliations:** 1 Departamento de Enfermería, Universitat de Valencia, 46010 Valencia, Spain; 2 Department of Psychology, Faculty of Education Sciences and Psychology, Universitat Rovira i Virgili, 43007 Tarragona, Spain; 3 Institut d’Investigació en Psicologia del RRHH, del Desenvolupament Organitzacional i de la Qualitat de Vida Laboral (IDOCAL), Universitat de Valencia, 46010 Valencia, Spain; aida.soriano@uv.es; 4 Escuela de Psicología, Universidad Adolfo Ibáñez, Campus de Peñalolén, Santiago de Chile 7941169, Chile; jesus.yeves@uai.cl

**Keywords:** Nursing, education quality, university education, satisfaction, education usefulness, undergraduates, job quality, mobility

## Abstract

The present study aimed to examine the relationship between the quality of undergraduate education perceived by novice nurses and their retrospective satisfaction with their education. It also studied the relationships between the perceived usefulness of their education for their current jobs and the quality of the jobs held by novice nursing professionals. The moderator role of mobility in this relationship was also analyzed, as it reflects a boundary condition in which additional preparation or job opportunities may occur. The study used data from the graduates’ survey carried out by the Agència per a la Qualitat del Sistema Universitari de Catalunya (AQU) in 2017. The analysis of data from 644 graduates of Catalan Universities in 2014 highlights different functions of two types of knowledge and skills; those directly related to science and the practice of nursing are stronger predictors of retrospective satisfaction with nursing education. In turn, the perception of the usefulness of horizontal skills, such as transversal and communication skills, plays a stronger role in predicting job quality. The results about the role of mobility were not conclusive, and more research is needed to clarify its influence on nursing education and subsequent professional practice.

## 1. Introduction

Traditionally, the university’s mission has been to teach and carry out scientific research oriented toward the generation, transfer, and dissemination of knowledge. However, this role has evolved as University education has grown in terms of the number of students and the demand for professional education, so that young people can obtain better employment and careers [1]. Universities play an intermediary role, contributing to better job placement in the knowledge economy, industry, and job markets [2]. This situation was summarized by Teichler [3] who stated “higher education rather than the pursuit of knowledge for its own sake, emphasizes extrinsic motivations of students and graduates, and focuses on the extent to which higher education is closely geared, as far as structures, curricula, knowledge bases, etc. are concerned, to employment and work” (p. 172). 

Consequently, not only is the long-term planning dimension assessed, giving coherence to curricula and universities, but students and employers are also well-equipped to judge the quality of certain aspects of higher education and the ability to perform workplace tasks [4]. In this regard, a large number of useful indicators measure and evaluate the quality of higher education. The Spanish university system has several public agencies that assess and guarantee the quality of the university system (e.g., “Agencia Nacional de Evaluación de la Calidad y Acreditación” (ANECA) or “Agència per a la Qualitat del Sistema Universitari de Catalunya” (AQU)). In the past decade new graduates have been surveyed periodically to obtain some indicators about their labor market entry and the quality of their jobs. These indicators have shown that the labor market has its own requirements in terms of the skills graduates must have to perform jobs. Despite the studies carried out, university educators and authorities only receive limited information and feedback about these competences and demands and thus the fit between education and human capital in labor markets is limited. As Nicolescu and Pun [4] pointed out, “there is still a gap between deliveries of higher education services and necessities of individuals and organizations […]. Obtaining employment after graduation is conditioned by having the abilities employers need and ask for” (p. 18). Qualifications and proficiency in relevant professional competences are critical in many disciplines, including nursing. Nurses need a wide range of competences to perform their work efficiently, and the requirements for these competences are changing [5]. Hence, the Organisation for Economic Co-operation and Development (OECD) [6] specifically pointed out that, when designing nursing education programs, it is crucial to make sure that future generations of nurses will have the right competences to be “fit for practice.” Moreover, important changes in the health area (e.g., pandemics, e-health, etc.; see [7]) are requiring learned information about the education–job demands that fit in this occupation. In this context, information and feedback from novice nurses about their studies may be helpful to further clarify this issue and its consequences. In addition, European programs have put a strong emphasis on mobility in University education. The European Union promotes mobility of future professionals through the Erasmus Program, granting about 10% of university students every year to study at least one semester in a university of another European Union country. Home universities recognize the credits and marks obtained in the host universities and include them in the students’ transcript of records. The program assumes that academic mobility is a good complement for professionals’ education, as it provides an opportunity for students to build up valuable competences [8]. Furthermore, in recent decades, nursing has become a profession with significant occupational mobility [9,10]. Job mobility across countries in Europe usually requires the nursing license credentials of the receiving country. In the case of Spain, the license requirements are the same all over the country, but this is not the case when mobility is to or from other European Union countries. Thus, the role of mobility in the relationship between quality of education and employability deserves research. The study of the quality and usefulness of nursing education to predict job quality of novice nurses in interaction with education and occupational mobility is needed to better understand the effectiveness of nursing education and their contribution to nurses’ career development.

The European Higher Education area, through the Bologna Agreement, proposed an educational model based not only on the acquisition of knowledge, but also on the development of competences. It also emphasized multidisciplinary education. Following the T-model of Service Science [11], pleads for integration of the vertical part of the T representing the competences in the specific professional field of the worker (e.g., nursing theoretical and practical competences), and the horizontal part (including knowledge from other related disciplines) [12]. Recently, Peiró and Soler [13] have added transversal competences (e.g., management of new technologies or relationships with colleagues and clients, teamwork, self-emotional regulation, conflict solving, and so on) in the horizontal axis. This extended model has proven useful to analyze the needs of future nursing education [7]. In sum, both types of knowledge and competences contribute to helping graduates to face the requirements of the labor market. The specific competences enhance their skills to perform the job role (as formally expected), and the other disciplines and the transversal ones help them to face the demands of the changing world in which we live. 

However, the implementation of this education model seems to present some problems in the current academic system. There is no doubt that traditional nursing pedagogies have produced efficient and knowledgeable nursing graduates for many years [14]. However, there is also empirical evidence suggesting that nursing education programs do not always produce competent and capable enough practitioners nowadays. Research has pointed out that a significant number of new nurses do not always have enough practical skills or the ability to apply their theoretical knowledge to practical situations [15,16,17,18]. This is especially evident in the transition process from student to registered nurse. There is no doubt that the transition from being a full-time student to becoming part of the workforce is a difficult period for many people [19], and it is especially difficult for nursing students because they have to face challenging situations that they may not be prepared to handle [20].

Accordingly, it seems necessary to assess and clarify the quality and usefulness of nursing education in current jobs from the young nursing professionals’ perspective. In the present study, quality of education is understood as the degree of excellence of the knowledge and competences acquired during their undergraduate studies, and the usefulness of the education for current job positions refers to how helpful the knowledge and skills acquired in nursing studies were in performing their job tasks.

The present study aims, first, to analyze the relationship between the education quality appraised by nursing graduates, and their retrospective satisfaction with the nursing education received; second, to explore the association between education usefulness for professional practice and the quality of their current jobs; and third to analyze the moderator role of mobility in the relationship between the education usefulness of nursing in current jobs and their job quality. In this way, this study contributes to reducing the gap between nursing education and human capital demands in this occupation by means of a deeper understanding of the nursing education components that influence retrospective satisfaction with their degree education and those components that predict quality indicators of their jobs.

### 1.1. Nursing Education Quality Appraised by Graduates after a Few Years of Nursing Experience and the Retrospective Satisfaction with Education 

There is a relevant amount of literature on the quality of education. Most studies based the quality of education concept on service quality, where quality is defined as the totality of features and characteristics of a service that affect its ability to satisfy expressed or implied needs [21]. This definition focuses on the customer and implies that services meet or exceed the needs, requirements, and expectations of the customers [22]. Thus, a service (e.g., education) that typically satisfies the needs of most of its customers (e.g., graduates) is a high-quality service (e.g., education). 

Contrast Theory [23] explains quality of service in terms of the consumer evaluation process after using a service, depending on the expectations and performance of services. If service performance exceeds expectations, consumers will feel quite satisfied; whereas if service performance is below expectations, consumers will be quite dissatisfied [24]. Hence, there is a strong association between quality of service appraised by costumers and customer satisfaction [25,26,27]. Customer satisfaction reflects the customer’s service evaluation compared to some type of reference such as the customer’s needs or expectations [28,29]. In terms of educational consumption, according to contrast theory, when the quality of the studies exceeds the expectations of the students, this has a positive impact on the satisfaction of the students with the education received. Based on this, the majority of studies about satisfaction in higher education reported that satisfaction resulted from the positive disconfirmation processes [30], that implies that student satisfaction can be derived from their experience and evaluation of the educational service provided [31].

Applied to higher education, people have multiple expectations about their education, such as opportunities for employment, career development, satisfaction, pleasure, and pride as students in college [32], but the most common and generalized expectation about education is probably the acquisition of both specialized knowledge and the necessary competences to succeed in a highly demanding job market [33]. Education (e.g., nursing) that satisfies these needs and expectations will probably be perceived as high-quality education, and graduates will be satisfied. In fact, some studies provide empirical support for this association [32,34]. However, this evaluation is context and time specific, and it is determined at the time when the evaluation occurs [35]. García-Aracil [36] using a European graduate sample, found that university provision and conditions (such as teaching quality, course content, design of degree, academic assistance, among others) positively influence graduates’ satisfaction with the university after their studies. Hence, it is important to understand the antecedents of the retrospective satisfaction with their studies of novice graduates with job experience. This provides important feedback for education institutions, teaching staff, and other relevant stakeholders. 

Accordingly, based on Contrast Theory [23,24], we examine this issue by proposing that graduates’ perception of the quality of the education received after they have work experience will be positively related to their retrospective satisfaction with the undergraduate nursing education received. Taking this into consideration, we formulate the following hypothesis:

**Hypothesis** **1.** *Quality of education, appraised after the graduates have work experience, will be positively related to retrospective satisfaction with the nursing education received*.

### 1.2. Nursing Education Quality and Job Quality

According to human capital theory [37,38,39], the efforts to develop knowledge, skills, and abilities (KSA), through factors like education and training, increase the individuals’ value to firms. This increase happens because the investment in KSA determines how well an individual performs [37]. Organizations acknowledge this higher value through higher pay and career progress [40]. Therefore, human capital represents the intrinsic value of employees’ knowledge and skills, and it is an important resource in obtaining a competitive advantage not only on the entry to the labor market, but also for employability in the longer term. Supporting this proposal, several studies have shown that the investment in human capital is not only related to extrinsic success indicators such as higher pay and higher status [41,42], but also to intrinsic ones such as job satisfaction [43]. Consequently, the investment in education helps individuals to achieve higher extrinsic and intrinsic job quality outcomes [44]. 

Job quality is a complex concept related to the job–worker relationship that considers both objective and subjective characteristics [45]. On the one hand, it includes objective indicators such as type of contract, full- and part-time job, or income; on the other hand, it also comprises subjective indicators such as job satisfaction [46]. Building on earlier work by González-Romá, Gamboa and Peiró [47], we conceptualized job quality in terms of four indicators: type of contract, full- and part-time job, salary, and job satisfaction.

Regarding nursing education, research has shown that the transition from student to registered nurse influences the quality of their jobs and their intention to leave a nursing job [48,49]. In addition, some research has examined the direct relationship between nursing education and job quality. For example, Kenny, Reeve, and Hall [50] showed the impact of educational satisfaction (work preparation component) on job satisfaction in a study of 204 registered nurses employed in their first job after graduating with bachelor’s degree in nursing. However, we are not aware of any study that has examined the relationship between nursing education, appraised from the perspective of its usefulness in the current job, and job quality, taking its multidimensionality into account.

Following the rationale of human capital theory [37,38], it seems plausible to suggest that the investment during higher education in skills that are useful for job performance will lead to a better job performance and, therefore, will be recognized and rewarded by employers. Consequently, we expect that perceived usefulness of nursing education in the current job will be related to job quality (e.g., type of contract, full- and part-time work, salary, and job satisfaction). Thus, the more their nursing education is perceived as useful in their job position, the higher the quality of nurses’ jobs will be. Therefore, we hypothesize that,

**Hypothesis** **2.** *The usefulness of education in the current job will be positively related to the type of contract (H2a), full- and part-time job (H2b), salary (H2c), and job satisfaction (H2d)*.

### 1.3. The Moderator Role of Mobility

Research supports the idea that academic and occupation geographic mobility favors the acquisition and development of knowledge and competences, which, in turn, has positive consequences in terms of job quality. According to human capital theory [37,38,39], mobility can also be considered a form of cumulating human capital because it provides access to a wider range of experiences through which individuals can acquire and develop knowledge, competences, abilities, and other personal characteristics. According to Bourdieu [51], students who move to study in an international arena, especially if they attend high prestigious universities, accumulate multiple and mutually-reinforcing forms of capital, such as human capital (a world-class university education), social capital (access to networks, ‘connections’), and mobility capital (“a subcomponent of human capital, enabling individuals to enhance their skills because of the richness of international experience gained by living abroad”) [52] (p.51). Then, educational mobility can be understood as an investment in one’s general human capital because it promotes foreign language proficiency as well as social, intercultural, and mobility skills [53], which goes along with the employers’ opinions that mobile students are more proactive and adaptable and are problem solvers [54]. On the other hand, following human capital theory, international work experiences after graduation have been considered as a specific form of human capital that provides valuable learning which has a positive impact on the long-term professional success of employees [55,56].

Consequently, mobility is a condition that may increase the probability of finding better-paid jobs (more than five years after graduation) [57,58]. Under this condition nurses may also find a good job more quickly [59], present higher employability [57], and advance more in their career [60], compared to people without mobility. More specifically, research has shown that mobility is an important motivational factor for nurses [61], and it provides greater access to on-the-job experience [62]. In addition, mobility is often an ‘added value’ to a graduate’s diploma, and it contributes to the acquisition of multiple competences [57]. Thus, we assume that mobility will boost the positive relationship between the perceived usefulness of undergraduate education for the job and the quality of the job held by the graduate.

**Hypothesis** **3.** *The relationship between the usefulness of education for the current job and the type of contract (H3a), full- and part-time job (H3b), salary (H3c), and job satisfaction (H3d) will be moderated by mobility, such that the relationship will be stronger when employees have experienced mobility*.

## 2. Materials and Methods

### 2.1. Study Design and Procedure

This study used the database of the Agència per a la Qualitat del Sistema Universitari de Catalunya (Agency for the Quality of the University System of Catalonia—AQU) regarding the workplace integration of graduates from Catalan universities from the perspective of graduates (AQU, 2017). Thus, this sample included graduates from all the public and private universities in Catalonia who graduated in any subject in the 2012–2013 academic year. They were surveyed in 2017. The total sample was composed of 15,563 people, representing 51.4% of the reference population, with a sampling error of 0.56%. In our study, we focused on graduates in Nursing (*n* = 670). We have chosen this degree because the rate of employment of the nursing graduates in a nursing job is close to 100% in the population studied. In fact, 96.1% (*n* = 644) of the nurses surveyed were working as nurses, and only 26 subjects were excluded because they didn’t fulfill this inclusion criterion. Nursing is then a suitable occupational group to study the relationships between education and nursing jobs’ quality of novice professionals. 

### 2.2. Variables and Measurement Tools

*Control variables.* Sex (0, woman; 1, man), age, and public/private organization (0 public; 1, private) were measured as control variables. 

*Education Quality (EQ-)*. It was measured with 10 items, with the following statement: “How would you rate the education you received at university?” The response scale ranged from 1 (very low) to 7 (very high). The 10 items were: (a) theoretical training; (b) practical training; (c) oral communication; (d) written communication; (e) teamwork; (f) leadership; (g) management; (h) problem solving; (i) making decisions; (j) creativity; (k) critical thinking; (l) computer science; (m) languages; and (n) documentation. 

*Education usefulness (EU-) in the current job*. It was measured with 10 items, with the following statement: “How useful is the university education you received in your current job?” The response scale ranged from 1 (very low) to 7 (very high). The 10 items were: (a) theoretical training; (b) practical training; (c) oral communication; (d) written communication; (e) teamwork; (f) leadership; (g) management; (h) problem solving; (i) making decisions; (j) creativity; (k) critical thinking; (l) computer science; (m) languages; and (n) documentation. 

*Mobility*. ‘Academic mobility’ is understood as the transferability of academic credits across European Union universities during the nursing studies, and ‘job mobility’ is the acceptance of a nursing license credential in other Spanish geographical areas. In other countries, mobility requires the fulfillment of a license in the receiving country. Mobility has been assessed with the following question: “Have you had any mobility experience?”, with the following response options: (a) no; (b) yes, during my degree (academic mobility); c) yes, job mobility; and (d) yes, academic and job mobility. This variable was codified as a dummy variable with two response options: (0) no, I haven’t; (1) yes, I have.

*Education satisfaction*. It was measured through a mono-item: “Rate from 1 (none) to 7 (many) your satisfaction with the usefulness of the knowledge from your degree for your work.”

*Type of contract*. This was assessed through the general statement: “What type of contract do you have or have you had?” The response options were: (1) permanent contract, (2) self-employed, (3) temporary contract, (4) intern, and (5) no contract. A frequencies analysis of this variable in our sample showed that 26.7% (*n* = 172) had permanent contracts, 0.8% (*n* = 5) were self-employed, 71.9% (*n* = 463) had temporary contracts, 0.5% (*n* = 3) were interns, and 0.2% (*n* = 1) had no contract. Taking these results into account, we focused on the general dichotomous response of temporary versus permanent employment, where intern was also considered a form of temporary employment, and self-employed and no-contract were codified as missing data. Thus, we codified a dummy variable with two response options: (0) temporary and (1) permanent contract.

*Full- and part-time work*. This was assessed through a general statement: “Do you have or have you had a full-time job?”, with two response options: (1) yes, and (2) no (part-time). Thus, it was codified as a dummy variable: (1) full-time job and (0) part-time job.

*Salary*. It was measured as the annual gross salary with the following response options: 1. Less than 12,000€; 2. 12,000–18,000€; 3. 18,001–30,000€; 4. 30,001–50,000€; and 5. More than 50,000€.

*Job Satisfaction*. This was assessed with a general statement, “Rate from 1 (none) to 7 (many) your satisfaction…”, and 4 items: 1. With the job content; 2. With the prospects of improvement and promotion; 3. With the remuneration; 4. With work in general. 

The design of the questionnaire and all the data gathering was run by the experts of the AQU (independently from the Universities involved), which is a public agency established by the Government with the aim of carrying out external quality assessment of the University education and promoting quality assurance.

### 2.3. Data Analysis

First, descriptive analyses were calculated to describe participant characteristics. Second, preliminary analyses were computed to further validate the measures used in this study: confirmatory factorial analysis and reliabilities of measures, and to examine the possible common method variance. Cronbach’s alphas were computed to examine the reliabilities of our measures. This study measured several variables through 7-point Likert scales, which could provoke common method variance [63]. Hence, we used Harman’s single-factor test with confirmatory factor analysis to assert that our research was not pervasively affected by common method variance. Likewise, descriptive statistics (means, standard deviations) and correlation analysis were computed. Third, for the first research objective, hierarchical multiple regression analyses were computed for the hypothesis testing. Fourth, the binary logistic regressions were computed to examine hypotheses related to type of contract and full- and part-time work as outcomes because they were dummy variables. Hierarchical multiple regressions were calculated to test the hypotheses for education satisfaction, salary, and job satisfaction. As Cohen and Cohen [64] described, first lower-order variables were introduced, and later the higher-order terms. We also conducted a simple slopes analysis to examine the interaction pattern [65]. Finally, in both logistic and hierarchical regressions, graphical representations were carried out to examine the interaction pattern [65], and centered scores were used to solve possible problems of multicollinearity. 

As all the hypotheses were directional and theory driven, we used one-tailed tests [66,67,68]. However, the use of one-tailed tests can increase the possibility of Type I errors and raises the possibility of threats to statistical conclusion validity. In this regard, we followed the guidelines provided by Kimmel [68] and Jones [66,67]. Jones [66] states, “a one-sided alternative is the most powerful test for all directional hypotheses, [therefore] it is strongly recommended that the one-tailed model be adopted wherever its use is appropriate” (p. 46). However, when the purpose is to determine whether a particular directional prediction is supported by the data, then “the one-tailed test is not only appropriate, but it is an error to use a two-tailed test model” [67] (p. 586). Thus, even though the possibility of Type I error can increase, we believe the use of one-tailed tests is appropriate for our research. Finally, an additional criterion was taken into account in testing the hypothesis to explain salary: working hours. Full-time salaries cannot be directly compared to part-time salaries because they are influenced by the number of working hours, producing a bias in the analysis. Accordingly, we only selected employees with a full-time contract (62%; *n* = 396) to examine the salary as a dependent variable. 

## 3. Results

### 3.1. Participant Characteristics

The total sample was composed of 15,563 people, representing 51.4% of the reference population, with a sampling error of 0.56%. In our study, we focused on graduates in Nursing (*n* = 670). An additional inclusion criterion was that subjects had to be working as nurses, which was the case for 96.1% (*n* = 644) of the sample, which means that 26 subjects were excluded. Finally, the sample consisted of 644 graduates in Nursing; 87% were women (*n* = 563), and 13% were men *(n* = 81). The average age was 27.75 years (standard deviation (SD) = 5.27); 94% were employed (*n* = 604), and 6% were unemployed at the time of the interview, but all of them had previously worked as nurses (*n* = 40). It is worth noting that the AQU survey indicated that, in the case of unemployment, answers should refer to the last job held. Among employees, 62% worked full-time (*n* = 396), and 38% worked part-time (*n* = 245); 72% had a permanent contract (*n* = 466), and 27% had a temporary contract (*n* = 172). Finally, 86.2% were Spanish, 2% came from other European countries (Erasmus mobility not computed), and 11.8% came from other continents, mainly Latin America.

### 3.2. Preliminary Results

Four confirmatory factorial analysis models were tested to validate the measures of quality of education and in a similar way the four models were tested for the education usefulness in the current job: a one-factor solution (M1); a three-factor solution (M2; knowledge and skills about nursing content; transversal competences; instrumental competences); a four-factor solution (M3; knowledge and skills about nursing content, communication competences, transversal competences, instrumental competences); and an alternative four-factor solution (M4; theoretical knowledge about nursing content, practice skills about nursing, transversal competences, instrumental competences). In addition, two models were tested for job satisfaction: a one-factor solution (with all the items) and a two-factor solution (considering intrinsic and extrinsic satisfaction). Table 1 presents the fit indexes of all these confirmatory factor analyses of the study measures (Table 1). Results presented a better fit of the four-factor solution for the measures both of quality of education and usefulness of education in the current job (M3a and M3b), compared to the other models. These four-factor models presented an excellent fit, except for the chi-squared Goodness-of-Fit Index, but this was probably due to the sample size (χ^2^ = 509.36, *p* < 0.01 for quality of education; χ^2^ = 255.12, *p* < 0.01 for usefulness of education in the current job). The Root Mean Square Error of Approximation (RMSEA) were 0.07 and 0.06, respectively, and showed a good fit because their value was lower than 0.08 [69]. For Comparative Fit Index (CFI) (0.95; 0.96), Incremental Fit Index (IFI) (0.95; 0.96), and Normed Fit Index (NFI) (0.93; 0.95), the values exceeded 0.90, also indicating a good fit [70]. By contrast, the other solutions showed a poorer fit between the data and the hypothesized model. The chi-squared Goodness-of-Fit Index (χ^2^) did not indicate a good fit, although, as mentioned above, this is probably due to the sample size. RMSEA indicated a value greater than or equal to 0.08, showing an inadequate or poor fit [69]. CFI, IFI, and NFI values also showed an unsatisfactory or poorer fit because they were below those obtained by the alternative four-factor solution or even below 0.90 [70]. In what concerns job satisfaction, the indexes showed a better fit of the one-factor model (CFI = 0.94; IFI = 0.94; NFI = 0.93; RMSEA = 0.16) compared to the two-factor solution (CFI = 0.90; IFI = 0.90; NFI = 0.90; RMSEA = 0.17). Thus, we used one general job satisfaction score.

The factor loading for all items ranged from 0.47 to 0.88; thus, they all exceeded the recommended level of 0.40 [71]. Table 2 presents the factor loadings of the measurement items.

To examine scale reliability, Cronbach’s alphas were computed. Reliabilities of all measures were acceptable because they exceeded or were near the cut-off value of 0.70 [72]. Table 2 shows the reliabilities values in the diagonal of correlation matrix. Finally, to assess potential common method variance, Harman’s one-factor test with confirmatory factor analysis was conducted. The results showed that the single-factor measurement model presented a poor fit (χ^2^ = 6603.65; df = 496; *p* = 0.00; CFI = 0.58; Tucker Lewis Index (TLI) = 0.53; NFI = 0.56; RMSEA = 0.14). Hence, common method variance did not seem to be a major problem for the interpretation of the results of this study [63]. 

Descriptive statistics (means and standard deviations) are presented in Table 2. Novice nurses rated the quality of their education as moderately high. More specifically, they more positively perceived knowledge and skills related to the specific nursing content (mean = 5.49; SD= 1.00), followed by communication skills (mean = 5.07; SD = 1.26), transversal skills (mean = 4.88; SD = 1.18), and finally instrumental skills (mean = 4.12; SD = 1.34). Regarding the usefulness of nursing education in their current jobs, novice nurses also assessed it as moderately high. The assessment of the usefulness of content, communication, and transversal skills was quite similar. Nevertheless, nurses perceived that content knowledge and skills were the most useful (mean = 5.48; SD = 1.14), followed by communication skills (mean = 5.30; SD = 1.19), transversal skills (mean = 5.26; SD = 1.11), and finally instrumental skills (mean = 4.54; SD = 1.35). Finally, correlations between education quality and retrospective satisfaction with the undergraduate education received were significant and positive (Table 3). Most of the correlations among the variables involved in the analysis of these relations were significant in the expected direction (Table 3).

### 3.3. Hypothesis Testing

#### 3.3.1. Quality of Education for the Job and Retrospective Satisfaction with the Usefulness of Undergraduate Education

Table 4 shows multiple hierarchical regression results for quality of education in predicting retrospective satisfaction with education, after controlling for sex and age. Results showed significant and positive relationships between EQ-nursing content and education satisfaction (β = 0.41, *p* < 0.01; one-tailed test). Nurses who perceived higher quality in the content component of undergraduate education presented greater satisfaction with the education received once they were working. Accordingly, Hypothesis 1 was partially supported.

In addition, significant direct relationships were found between some control variables (sex and age) and retrospective satisfaction with education. Women experienced higher levels of education satisfaction than men, and older nurses had higher levels than younger ones.

#### 3.3.2. Job Quality as an Output of the Usefulness of Undergraduate Education

Table 5 presents regression results for usefulness of the different components of the education for the current job and mobility in predicting the indicators of quality of work insertion (type of contract, full- and part-time work, salary, and job satisfaction), after controlling for sex and age. Binary logistic regressions were computed to predict the type of contract and full- and part-time work. The chi-squared statistics for the models were statistically significant, pointing out that the null hypothesis should be rejected. In fact, the models in Step 3 presented a significant chi-squared increase over the previous models (∆χ^2^ (12) = 7, *p* < 0.01 for type of contract; ∆χ^2^ (12) = 4.76, *p* < 0.01 for full- and part-time work). Odds ratios greater than 1 reflect the non-reference category, and odds ratios below 1 suggest reduced odds, in the case of variables with two categories.

Results partially supported Hypothesis 2. EU-communication skills were positively related to type of contract (B = 0.545, Wald (1) = 2.72, *p* < 0.05; one-tailed test), whereas EU-transversal skills were negatively associated with it (B = −0.841, Wald (1) = 4.15, *p* < 0.05; one-tailed test). The probability of having permanent employment is greater for nurses with a higher level of communication skills. In contrast, the probability of having temporary employment is greater for nurses with higher levels of transversal skills (H2a). 

EU-communication skills were positively related to full-time work (B = 0.493, Wald (1) = 3.60, *p* < 0.05; one-tailed test). The probability of having a full-time job was higher for nurses with higher levels of communication skills (H2b). EU-communication skills were also positively related to salary (β = 0.211; *p* < 0.05; one-tailed test); thus, nurses with higher levels of communication skills had higher salaries (H2c). 

EU-instrumental skills were positively related to job satisfaction (β = 0.208; *p* < 0.05; one-tailed test). Nurses with higher levels of instrumental skills experienced higher levels of job satisfaction. Finally, a negative direct relationship between mobility and job satisfaction was found (β = −0.073; *p* < 0.05; one-tailed test). Nurses with mobility experienced lower job satisfaction than those without mobility (H2d). 

**Figure 1:**
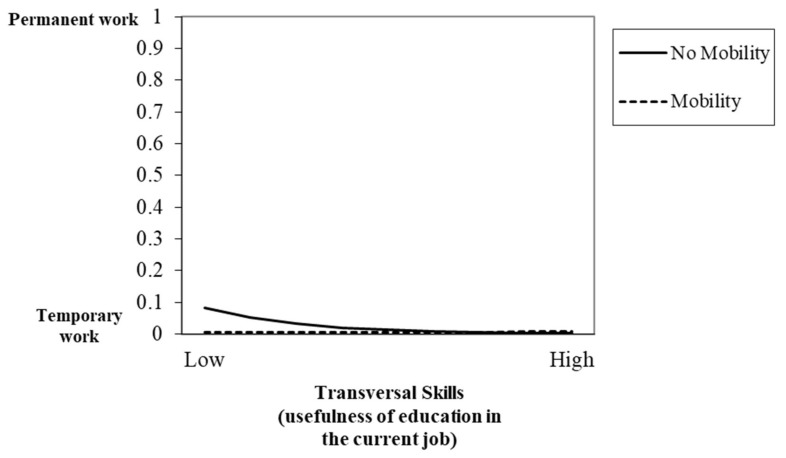
Interaction between transversal skills (usefulness of education in the current job) and mobility in predicting type of contract.

**Figure 2:**
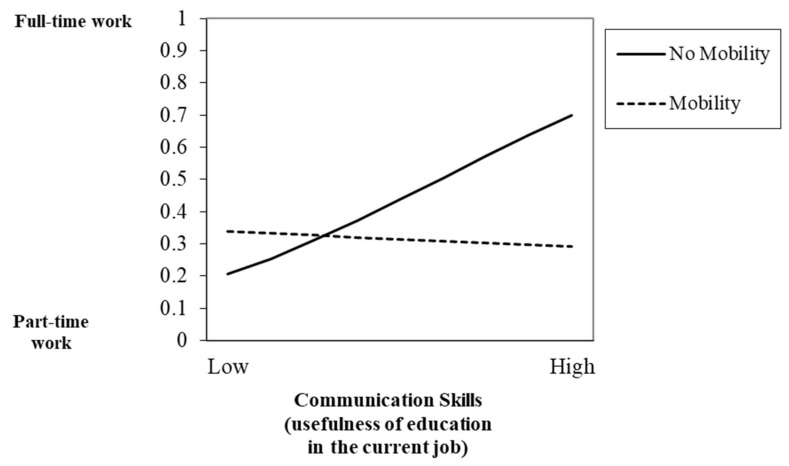
Interaction between communication skills (usefulness of education in the current job) and mobility in predicting full- and part-time work.

Hypothesis 3, which proposed the moderator role of mobility in the relationship between education usefulness in the current job and job quality, was also partially supported. Results showed significant interaction effects between education usefulness in the current job and mobility in predicting quality of work insertion. Figure 1 displays the interaction effect of EU-transversal skills and mobility in predicting type of contract. Nurses had a similar probability of having temporary employment, regardless of their mobility or lack of it, when they had high levels of EU-transversal skills. However, when their EU-transversal skills were low, nurses without mobility had a higher probability of holding permanent jobs (H3a). Regarding full- and part-time work, Figure 2 displays the interaction effect between EU-communication skills and mobility. Nurses with low EU-communication skills and mobility presented a higher probability of having full-time jobs, compared to nurses without any mobility experience. However, there was a slight decrease in the probability of having full-time work when nurses had high EU-communication skills and mobility. There was also an increase in the probability of having full-time work when nurses had higher levels of EU-communication skills but no mobility (H3b).

**Figure 3:**
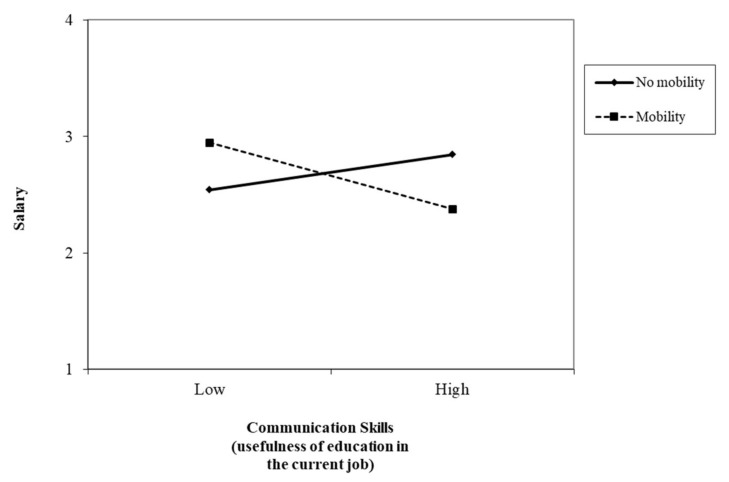
Interaction between communication skills (usefulness of education in the current job) and mobility in predicting salary.

**Figure 4:**
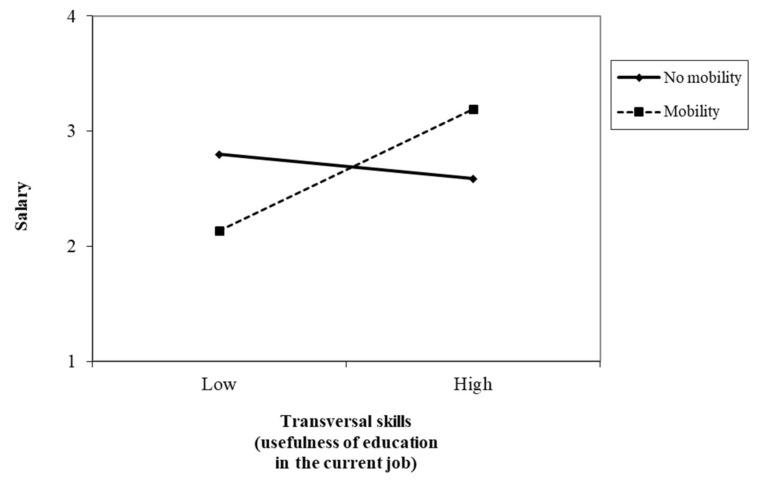
Interaction between transversal skills (usefulness of education in the current job) and mobility in predicting salary.

Figure 3 shows the interaction effect of EU-communication skills and mobility in predicting salary. Nurses with high EU-communication skills presented higher salaries when they had no mobility. The simple slopes of the regression of salary onto EU-communication skills with mobility (β = −0.29, t = −2.61, *p* = 0.01; one-tailed) and no mobility (β = 0.15, t = 3.38, *p* = 0.00; one-tailed) were significant. In contrast, nurses with high EU-transversal skills had higher salaries when they had mobility, compared to nurses with no mobility, whose salaries were lower (Figure 4). The simple slopes of the regression of salary onto EU-communication skills with mobility (β = 0.53, t = 4.82, *p* = 0.00; one-tailed) and no mobility (β = −0.10, t = −2.34, *p* = 0.02; one-tailed) were significant (H3c).

**Figure 5:**
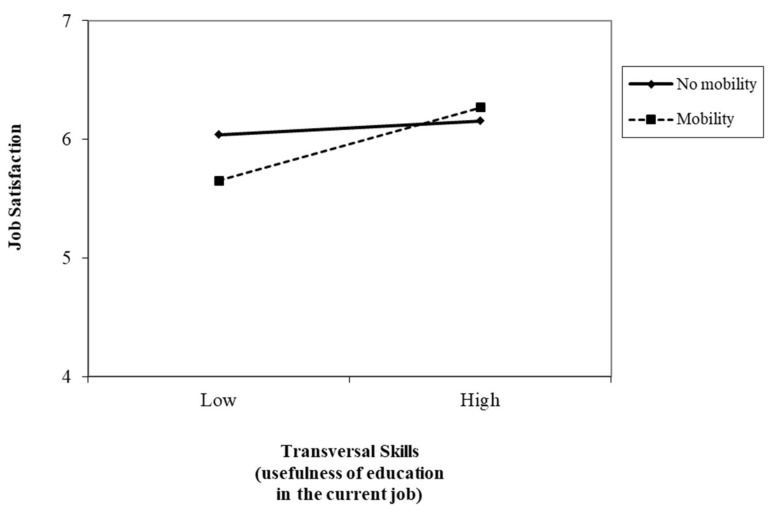
Interaction between transversal skills (usefulness of education in the current job) and mobility in predicting job satisfaction.

Figure 5 displays the interaction effect of EU-transversal skills and mobility in predicting job satisfaction. The simple slopes of the regression of job satisfaction onto EU-transversal skills with mobility were significant (β = 0.31, t = 2.82, *p* = 0.00; one-tailed), but not in the case of no mobility (β = 0.05, t = 1.29, *p* = 0.19; one-tailed). Therefore, nurses with high levels of EU-transversal skills experienced higher job satisfaction when they had mobility. However, job satisfaction levels were similar, regardless of the level of EU-transversal skills, when nurses had no mobility (H3d).

In addition, the control variables presented significant direct effects. The relationship between sex and job satisfaction was significant and negative (β = −0.159; *p* < 0.05; one-tailed test). Men reported lower levels of job satisfaction than women. Age was positively related to full-time and type of contract (B = 0.094, Wald (1) = 25.05, *p* < 0.01; one-tailed test) and job satisfaction (β = 0.07; *p* < 0.01; one-tailed test). Older nurses had a greater probability of being permanent than younger nurses. They also presented higher job satisfaction than younger nurses. Ownership was also significantly related to type of contract (B = 0.92, Wald (1) = 19.26, *p* < 0.01; one-tailed test), salary (β = −0.21; *p* < 0.05; one-tailed test), and job satisfaction (β = −0.18; *p* < 0.05; one-tailed test). Private organizations presented a higher proportion of permanent nurses, and nurses who worked in them received lower salaries and experienced lower levels of job satisfaction compared to nurses in public organizations.

## 4. Discussion

The present research aimed to examine the quality and usefulness of nursing education in current jobs in Catalonia. Furthermore, it analyzed the relationship between the quality of nursing education and the retrospective satisfaction with this education when working as a nurse. Finally, we studied the relationship between the usefulness of their nursing education in the current job and the job quality, as well as the moderator role of mobility in this relationship.

This study contributed to clarifying how nurses who graduated approximately three years earlier perceived nursing education once they were working. Overall, young nurses appraised their education as moderately high in terms of quality and usefulness for their current jobs. Therefore, recently graduated nurses in Catalonia acquire, on average, good and useful education during their nursing education to perform their job. These results show the importance of both specific professional knowledge and skills and transversal skills, according to the T-model of professional education [13]. The present study validated this model by showing that not only vertical (or specific) knowledge and skills, but also horizontal skills, enhance professionals’ value in the job market.

The second contribution is related to the relationships identified between the quality of the undergraduate education received and the retrospective satisfaction with that education. Results showed that the positive appraisal of the quality of nursing education was significantly related to education satisfaction. However, when controlling the effects of all the dimensions of the education considered, only specific knowledge and skills for nursing had significant predictive power. In other words, when the different education components are analyzed together, only content knowledge and skills predict retrospective nurses’ satisfaction with their undergraduate education, showing the main role of the vertical (or specific) competences in education satisfaction, according to the T-model [13]. This result could also be explained by the retrospective approach of our study and the success criteria that still prevail in work contexts, where competences are mainly understood as knowledge and skills in the topics directly related to professional practice. More attention should be paid to the horizontal or transversal competences and skills in professional performance.

Regarding the relationship between the perceived usefulness of nursing education for the current job and the job quality indicators for this job, the results showed: first, that the probability of achieving permanent employment is greater for nurses who perceive a higher level of usefulness of the communication and transversal skills acquired during undergraduate education; second, that the odds of having full-time jobs and higher salaries were higher for nurses who perceived a higher level of usefulness of the communication skills learned during their studies; and third, that nurses who perceived a higher level of usefulness of instrumental skills experienced higher levels of job satisfaction. In this regard, the usefulness of the education in horizontal skills (transversal, communicative, and instrumental) during undergraduate education is important in obtaining higher quality jobs after graduation. Overall, these results are congruent with previous studies that demonstrate the importance of acquiring transversal skills during nursing studies in order to cope with the demands of today’s changing job market [5,33]. The role of communication skills is highlighted because their usefulness contributes to positively and significantly predicting every objective indicator of job quality (e.g., type of contract, full- and part-time, and salary). Communication is a core component of many nursing jobs, and its mastery provides valuable human capital for nursing [9,10].

Finally, the results provide insight into the moderator role of mobility in the relationship between the usefulness of nursing education in the current job and job quality. Although previous literature supported the hypothesis that mobility would enhance the positive effects of nursing education’s usefulness on job quality, the results showed that this moderator effect was only present for transversal and communication skills. In accordance with human capital theory [37,38,39], mobile nurses who perceived high usefulness of transversal skills held jobs with higher salaries and experienced greater job satisfaction than those who had not experienced any mobility for education and/or job purposes. However, contrary to human capital theory [37,38,39], the results showed that mobile nurses who perceived a high usefulness of the communication skills learned during their studies held lower quality jobs, in terms of part-time work and a lower salary, compared to non-mobile nurses. Likewise, mobile nurses who perceived a greater usefulness of transversal skills presented slightly lower job quality because their odds of having temporary contracts were somewhat higher. In sum, mobility only seems to promote job quality in terms of salary and job satisfaction. Taking into consideration the other criteria (type of contract and full-time work), it may play a detrimental role in job quality. These contradictory results are consistent with the study by Brown and Jones [62], which states that “although anecdotal evidence suggests the value of geographical mobility in obtaining senior nursing positions in Australia, there has been little research on whether geographical mobility does affect the chances of being in a senior position” (p. 4). A possible explanation for these inconsistent results could be based on the particularities of health systems across countries, and several mobility features should be disentangled (duration of mobility, type, purpose, etc.). In this regard, a recent study [73] showed the critical role of global ‘structures of prestige’ of higher education systems and labor markets in university students’ mobility. These authors suggest that students move to countries that are higher or lower ranked in terms of labor markets and higher education systems, and this is associated with different career outcomes. Similarly, it is possible that, due to different particularities of health systems, employers may more favorably evaluate those who have developed their competences in the same country, thus providing them with better job quality. The effect of mobility in nursing does not seem to be conclusive during their education and professional careers. Hence, more research is needed to better understand the role mobility plays in nurses’ job quality.

### 4.1. Limitations

Our study has several limitations to keep in mind when interpreting the results. First, causal relationships between variables cannot be inferred because a cross-sectional design was used. Longitudinal research is necessary to further investigate the causal relationships. Second, all our variables were measured with self-reported questionnaires, and so there can be a risk of common method bias. In this respect, we computed Harman’s one-factor test to assess common method variance and results showed that it was not a threat in the interpretation of the results of this study. Nevertheless, it is also recommendable that other methods could usefully be applied in future research to provide further evidence about the relationships found here. Third, the measure of mobility combined both academic and job mobility. It is possible that different types of mobility have different outcomes. Future studies might consider them separately to analyze their possible differences. In addition, other features of mobility should be differentiated (e.g., duration, destination, etc.) to disentangle the inconsistent results obtained. Finally, this study focused on nursing education. However, it seems plausible to think that other education programs would also benefit from this type of studies. Hence, we recommend broadening the study focus and examining education quality and usefulness and their work consequences for young professionals in other disciplines.

### 4.2. Theoretical and Practical Implications

This study has relevant theoretical implications. First, these results are in line with the postulates of the contrast theory [23], showing how the perception of the quality of a service impacts on satisfaction. Specifically, in educational terms, these results contribute showing how the evaluation of the quality of the education received, especially the specific content, influences the satisfaction of nurses’ graduates. Second, our results provide a deeper understanding of the contrast theory as it focuses on retrospective lagged-satisfaction evaluation and in that way provides additional evidence about the context and time-bounded features of satisfaction assessment. Moreover, it shows that the differentiated assessment of quality concerning different components of the education program play a differential predictive role on retrospective satisfaction once the graduate already held a job. Future studies will need to clarify changes in individuals’ criteria to assess education contents and outcomes from different perspectives of the students and graduates. In addition, our study contributes to human capital theory [37,38] by showing that the investment in qualifications and skills, both profession-specific and transversal, are important assets in obtaining high quality jobs (in terms of salary, contract, dedication, and job satisfaction). In this case, our results contribute by highlighting the importance that the usefulness of horizontal competences has to obtain a quality job and, therefore, should play an important role in human capital investment. Finally, we have presented mixed evidence about the role of mobility in the education-work link, showing that this role is more complex than the simple assumption that mobility is always an added value for nursing professionals and future research is needed to further clarify these issues.

In terms of practical implications, our results show the importance of the quality and usefulness of nursing education in current jobs, as well as their implications for recent nursing graduates when they enter the labor market. In this regard, our results have shown that nursing graduates in Catalonia, once they were working, on average appraised their undergraduate education as having quality and being useful for their current job positions. The results also showed that the quality of education, retrospectively assessed, was significantly and positively related to nurses’ overall lagged satisfaction with their education. One important contribution of our study is its feedback function to help universities to pinpoint their strengths and identify areas for improvement in nursing education. Results show how different educational aspects may contribute to obtaining greater satisfaction with nursing education or to finding good quality jobs. These results provide significant inputs for future improvements in the design of nursing education. For instance, universities may consider strengthening content knowledge, skills, and competences as they are critical for the lagged satisfaction of nursing graduates. Moreover, academic authorities should pay attention to enhance skills and communication competences in nursing education as they seem to be important to find a quality job. Finally, university vocational services should offer counseling to nursing students and graduates about developing and practicing all these key competences and skills and inform them on how these can be valuable in enhancing job quality.

## 5. Conclusions

The educational period is an important stage that provides the necessary knowledge and competences for professional nurses. The nursing curriculum will determine young nurses’ education satisfaction as well as their possibilities of getting a quality job. This study identified different knowledge, skills, and competences that are the most relevant in determining these outcomes. It highlights the importance of vertical (or professional) competences in education satisfaction and professional success, whereas the usefulness of horizontal skills and competences, such as transversal, communicative, and instrumental skills, plays an important role in obtaining a higher quality job, especially the communication competences. Finally, the role of mobility was examined, and, surprisingly, it showed an ambivalent and mixed role, with some criteria improving job quality, but others deteriorating it, when interacting with education appraisal. Further research is necessary to clarify the influence of mobility on nursing education and professional practice. These results have relevant practical implications for strengthening some aspects of nursing education and improving graduates’ satisfaction and quality job insertion.

## Figures and Tables

**Table 1:** Summary of confirmatory factor analyses: results of Education Quality (EQ-; models M1a–M4a) and Education Usefulness (EU-; Models M1b–M4b).

	Chi-Squared	gl	*p*	CFI	IFI	NFI	RMSEA
*Education Quality (EQ-)*							
One-factor model (M1a)	599.13	78	0.00	0.89	0.89	0.88	0.10
Three-factor model (M2a)	511.85	77	0.00	0.91	0.91	0.90	0.09
Four-factor model (M3a)	344.82	75	0.00	0.95	0.95	0.93	0.07
Alternative four-factor model (M4a)	509.36	75	0.00	0.91	0.91	0.90	0.09
*Education usefulness (EU-) in the current job*							
One-factor model (M1b)	599.50	78	0.00	0.89	0.89	0.87	0.10
Three-factor model (M2b)	439.04	77	0.00	0.92	0.92	0.91	0.08
Four-factor model (M3b)	255.12	75	0.00	0.96	0.96	0.95	0.06
Alternative four-factor model (M4b)	255.12	75	0.00	0.92	0.92	0.91	0.09
*Job Satisfaction*							
One-factor model (M5)	37.00	2	0.00	0.94	0.94	0.93	0.16
Two-factor model (M6)	56.88	3	0.00	0.90	0.90	0.90	0.17

M1a/M1b. One-factor model; M2a/M2b. knowledge and skills about nursing contents transversal skills, instrumental skills; M3a/M3b. knowledge and skills about nursing contents, communication skills, transversal skills, instrumental skills; M4a/M4b. theoretical training, practical training, transversal skills, instrumental skills; M5. One-factor model; M6. Intrinsic satisfaction, extrinsic satisfaction. Gl, Degrees of freedom; CFI, Comparative Fit Index; IFI, Incremental Fit Index; NFI, Normed Fit Index; RMSEA, Root Mean Square Error of Approximation.

**Table 2:** Factor loading matrix.

		Education Quality (EQ)				Education Usefulness (EU)				Satisfaction
		1	2	3	4	1	2	3	4	1
Nursing content	Theoretical training	0.83				0.80				
	Practical training	0.63				0.71				
Communication skills	Oral communication		0.77				0.72			
	Written communication		0.88				0.82			
Transversal skills	Teamwork			0.76				0.78		
	Leadership			0.83				0.77		
	Management			0.78				0.75		
	Problem solving			0.84				0.84		
	Making decisions			0.84				0.85		
	Creativity			0.76				0.70		
	Critical thinking			0.71				0.70		
Instrumental skills	Computer science				0.65				0.64	
	Languages				0.63				0.61	
	Documentation				0.69				0.71	
Satisfaction	With the job content									0.73
	With the prospects of improvement and promotion									0.49
	With the remuneration									0.47
	With work in general									0.84

**Table 3:** Descriptive statistics (means and standard deviations) and correlations.

	Mean	SD	*a*	1	2	3	4	5	6	7	8	9	10	11	12	13	14	15	16	17
1. Sex	−	−	−	1.0																
2.Age	27.75	5.27	−	0.09 ^*^	1.0															
3.Public/private organization	−	−	−	−0.04	0.05	1.0														
4. EQ-Nursing Content	5.49	1.00	0.72	−0.10 ^**^	0.08 ^*^	0.01	1.0													
5. EQ-Communication skills	5.07	1.26	0.79	−0.11 ^**^	0.13 ^**^	0.01	0.52 ^**^	1.0												
6. EQ-Transversal skills	4.88	1.18	0.91	−0.17 ^**^	0.08 ^*^	−0.01	0.58 ^**^	0.67 ^**^	1.0											
7. EQ-Instrumental skills	4.12	1.34	0.72	−0.09 ^**^	0.17 ^**^	0.03	0.45 ^**^	0.56 ^**^	0.69 ^**^	1.0										
8. Education satisfaction	5.48	1.35	−	−0.14 ^**^	0.10 ^*^	−0.04	0.50 ^**^	0.33 **	0.40 **	0.32 **	1.0									
9. EU-Nursing Content	5.48	1.14	0.63	−0.07 ^*^	0.07 ^*^	−0.04	0.75 ^**^	0.41 ^**^	0.51 ^**^	0.37 ^**^	0.56 **	1.0								
10. EU-Communication skills	5.30	1.19	0.83	−0.06 ^*^	0.10 ^**^	0.00	0.50 ^**^	0.69 ^**^	0.55 ^**^	0.46 ^**^	0.38 **	0.56 ^**^	1.0							
11. EU-Transversal skills	5.26	1.11	0.91	−0.08 ^*^	0.05	−0.03	0.53 ^**^	0.43 ^**^	0.70 ^**^	0.47 ^**^	0.37 **	0.59 ^**^	0.62 ^**^	1.0						
12. EU-Instrumental skills	4.54	1.35	0.72	−0.04	0.11 ^**^	−0.01	0.41 ^**^	0.36 ^**^	0.49 ^**^	0.60 ^**^	0.37 **	0.44 ^**^	0.49 ^**^	0.66 ^**^	1.0					
13. Mobility	−	−	−	0.03	−0.07 ^*^	−0.07	−0.01	−0.05	0.09 ^**^	0.10 ^**^	−0.03	−0.01	−0.01	0.07 ^*^	0.08 ^*^	1.0				
14. Type of contract	−	−	−	0.03	0.24 ^**^	0.21 ^**^	0.03	0.04	0.04	0.12 ^**^	0.01	0.01	0.07 ^*^	0.04	0.06	0.02	1.0			
15. Full- and part-time work	−	−	−	−0.13 ^**^	0.09 ^*^	0.02	0.49 ^**^	0.32 ^**^	0.39 ^**^	0.32 ^**^	0.11 **	0.56 ^**^	0.37 ^**^	0.37 ^**^	0.37 ^**^	−0.03	0.15 **	1.0		
16. Salary	2.59	0.71	−	0.065	0.036	−0.11 *	0.01	0.08	0.08	0.03	0.04	0.04	0.04	0.07	0.05	−0.05	0.09	−	1.0	
17. Job satisfaction	5.48	0.87	0.70	−0.13 ^**^	0.05	−0.17 ^**^	0.16 ^**^	0.19 ^**^	0.20 ^**^	0.20 ^**^	0.21 **	0.18 ^**^	0.20 ^**^	0.22 ^**^	0.26 ^**^	−0.03	−0.01	0.13 **	0.06	1.0

* *p* < 0.05 ** *p* < 0.01, one-tailed. SD (Standard deviation). EQ- (Education Quality). EU- (Education usefulness). Note. Sex (0 woman; 1 men). Mobility (0. no; 1. yes). Type of contract: (0, temporary; 1 fixed). Full- and part-time work (1. Full-time; 0 part-time). All data were computed in the whole sample, except the results for salary, which were computed only in the full-time work sample. Content refers to knowledge and skills about nursing.

**Table 4:** Regression analysis of graduates’ retrospective Education Satisfaction on Education Quality (EQ-) factors.

	Education Satisfaction		
	B	SE	*p*
Step 1			
Sex	−0.157	0.180	0.000
Age	0.111	0.011	0.012
Public/private organization	−0.050	0.121	0.258
Step 2			
EQ-Nursing content	0.409	0.066	0.000
EQ-Communication skills	0.007	0.081	0.895
EQ-Transversal skills	0.100	0.090	0.112
EQ-Instrumental skills	0.046	0.073	0.386
R^2^	0.278		
R^2^ change step 1	0.034		0.000
R^2^ change step 2	0.244		0.000

B are standardized values. Given that *p*-values were computed as two-tailed, they must be divided by 2 to obtain the *p*-value for the one-tailed test. Sex (0 woman; 1 men). Public/private organization (0. public; 1. Private). B, standardized coefficient; SE, standard error; *p*, *p*-value. R^2^, R-squared.

**Table 5:** Regression analysis for Education Usefulness (EU-) in current job and mobility in predicting salary, job satisfaction, full/part-time, and type of contract.

	Type of Contract			Full- and Part-Time work				Salary				Job Satisfaction		
	B	Wald	*p*	Exp(B)	B	Wald	*p*	Exp(B)	B	SE	*p*	B	SE	*p*
Step 1														
Sex	−0.106	0.117	0.732	0.899	0.399	20.154	0.142	10.491	0.066	0.166	0.475	−0.159	0.110	0.000
Age	0.094	25.051	0.000	1.099	0.029	2.456	0.117	1.029	0.077	0.009	0.402	0.073	0.007	0.093
Public/private organization	0.925	19.264	0.000	2.523	0.043	0.053	0.818	1.044	−0.213	0.123	0.022	−0.187	0.074	0.000
Step 2														
EU-Nursing Content	0.084	0.082	0.775	1.087	0.086	0.128	0.721	1.090	0.120	0.095	0.345	0.027	0.047	0.610
EU-Communication skills (CS)	0.545	2.723	0.099	1.724	0.493	3.602	0.058	1.637	0.211	0.086	0.080	0.018	0.050	0.751
EU-Transversal skills (TS)	−0.841	4.157	0.041	0.431	−0.377	1.132	0.287	0.686	−0.149	0.108	0.332	0.063	0.059	0.326
EU-Instrumental skills (IS)	0.434	2.031	0.154	1.543	0.197	0.578	0.447	1.218	0.134	0.093	0.272	0.208	0.050	0.000
Mobility	−0.360	2.367	0.124	0.698	−0.262	1.556	0.212	0.769	−0.023	0.135	0.814	−0.073	0.079	0.082
Step 3														
Content *mobility	−0.194	0.325	0.569	0.824	0.248	0.775	0.379	1.281	0.118	0.213	0.514	−0.047	0.108	0.476
CS *mobility	−0.435	1.328	0.249	0.647	−0.600	3.983	0.046	0.549	−0.364	0.231	0.062	−0.081	0.114	0.229
TS *mobility	1.074	5.450	0.020	2.928	0.374	0.908	0.341	1.453	0.516	0.281	0.026	0.132	0.155	0.107
IS *mobility	−0.529	2.254	0.133	0.589	−0.152	0.256	0.613	0.859	−0.265	0.199	0.148	−0.039	0.116	0.566
R^2^	0.150 ^a^				0.049 ^a^				0.186			0.143		
R^2^ change step 1/χ2(gl)	47.40 (3)		0.000		5.38 (3)		0.146		0.058		0.078	0.060 **		0.000
R^2^ change step 2/χ2(gl)	52.63 (8)		0.000		15.45 (8)		0.051		0.072		0.021	0.077 **		0.000
R^2^ change step 3/χ2(gl)	59.04 (12)		0.000		20.21 (12)		0.063		0.055		0.142	0.006		0.498

Note. Given that *p*-values were computed as two-tailed, they must be divided by 2 to obtain the *p*-value for the one-tailed test. R^2^ change step refers to multiple hierarchical regression and χ^2^ to binary logistic regressions. Sex (0 woman; 1 men). Public/private organization (0. public; 1. Private). Mobility (0. no; 1. yes). Type of contract: (0. temporary; 1. permanent). Full/part-time work (0, part-time; 1, full-time). ^a^ It is Nagelkerke’s R^2^ because it is a logistic regression. Content refers to knowledge and skills about nursing. B, standardized coefficient; SE, standard error; *p*, *p*-value; EU, Education usefulness; CS, Communication skills; TS, Transversal skills; IS, Instrumental skills; Exp(B), exponentiation of the B coefficient.
